# Climate-neutral and Smart Cities: a critical review through the lens of environmental justice

**DOI:** 10.3389/fsoc.2023.1175592

**Published:** 2023-10-03

**Authors:** Ilaria Beretta, Caterina Bracchi

**Affiliations:** Postagraduate School for Environmental Studies (ASA), Università Cattolica del Sacro Cuore, Brescia, Italy

**Keywords:** environmental justice, smart cities, green cities, social inclusiveness, Mission Climate -neutral and Smart Cities

## Abstract

The political choices made by the European institutions in the last twenty years show how the conviction is increasingly rooted that the management of environmental problems and, more specifically, the fight against climate change can find a valid solution in technology and eco-innovations. This is evident starting from the last two growth strategies adopted (Europe 2020 and the European Green Deal), from the long series of measures implemented to put them into practice and from the main R&I funding programs, such as Horizon Europe. In this context, the problem of justice and inclusiveness of the various initiatives implemented is attracting growing attention. In fact, if the institutional documents assume that green and smart participated projects are also fair and inclusive, a growing body of literature based on empirical studies seems to refute this assumption. Within this framework, the present work analyses first the critical literature and then the three main preparatory documents for the Horizon Europe Mission Climate-neutral and Smart Cities, which selected 100 European cities to become climate-neutral by 2030. These have been studied through the lens of environmental justice, in order to assess the European Commission’s understanding of the existing and arising equity issues in the path toward climate neutrality. The research shows that, while the first two documents seemed informed by the idea that participation automatically translates into equality, the last guidelines show a deeper acknowledgement of the multidimensional nature of environmental justice. One that, beyond participation, also considers issues of distribution, rights, responsibilities and recognition. The present work should nevertheless be understood as a preparatory, analytical tool that will require the further definition and implementation of Climate City Contracts by the selected cities, in order to assess how the issue of environmental justice is effectively being considered in each specific context.

## 1. Introduction

The political choices made by the European institutions in the last twenty years show how the conviction is increasingly rooted that the management of environmental problems and, more specifically, the fight against climate change can find a valid solution in technology and eco-innovations. This is evident starting from the last two growth strategies adopted (Europe 2020^2^ and the European Green Deal^3^), from the long series of measures implemented to put them into practice and from the main R&I funding programs, such as Horizon Europe (see infra). At the same time, these strategies and programs present themselves as participatory, just and inclusive. This can be found, for example, in the first lines of the European Green Deal: ‘It also aims to protect, conserve and enhance the EU’s natural capital, and protect the health and well-being of citizens from environment-related risks and impacts. At the same time, this transition must be just and inclusive’ [COM(2019) 640 final, p. 2].

In this context, cities are increasingly assuming a key role, as political and economic players, in supporting the achievement of sustainability objectives also through green initiatives (Thorns, 2002; Betsill and Bulkeley, 2006). Cities, for example, are required to adopt mitigation and adaptation plans against climate change, thus reducing its negative impacts on individuals, infrastructure and resources (Portney, 2013). At the same time, however, cities are also expected to represent the main growth drivers of the global economy, proposing win-win solutions that allow the growth of liberal economies and environmental protection at the same time (Anguelovski and Martínez Alier, 2014).

In the face of these institutional choices which highlight how the European Union – in the great challenge toward climate neutrality – believes, on the one hand, in the ‘salvific properties’ of technologies and, on the other, in the inclusiveness of their impacts, there is a growing literature, above all from environmental justice and urban political ecology, questioning this belief and demonstrating how greening can be an essential tool in the struggle for development and capital accumulation in urban contexts (Checker, 2011; Gould and Lewis, 2012; Bryson, 2013). Critical studies show that “urban greening is a deeply political project grounded in technocratic principles and the naive apolitical assumption that greening will, unassisted, result in both more just and prosperous cities” (Anguelovski et al., 2020, p. 1745).

In order to address how environmental justice is taken into account in the latest European strategy for Climate-neutral and Smart cities, the present work is structured as follows. First, the main sociological reference literature will be reviewed in order to build the theoretical framework, which will be used in the analytical part in paragraphs three and four. The first paragraph is devoted to smart cities literature and their main critical aspects, while the second focuses on the environmental sustainability and justice discourses. The third paragraph will then be devoted to introducing what could be identified as the European Commission’s strategy toward climate neutrality for European cities, the Horizon Europe Mission Climate-neutral and Smart Cities. Building on the literature review, and applying the environmental justice definition developed by Bulkeley et al. (2014), the fourth paragraph will then propose an analysis of the Mission through the lens of environmental justice as a multidimensional concept. Finally, in the conclusions, an attempt will be made to evaluate the acknowledgment of issues of environmental justice in the Cities Mission, with a proposal for further research.

### 1.1. Smart cities and inclusiveness

The idea of “smart cities” has gained remarkable popularity over the last years (de Jong et al., 2015; Michalec et al., 2019), becoming a buzzword that impregantes urban narratives around sustainability, liveability, low-carbon, green growth and urban efficiency. Technologies such as sensors, smart grids, smart meters, big data, integrated platforms lie at the heart of many smart urban interventions to combat global environmental crises and climate change (March, 2021). Although there are different definitions (Beretta, 2015), which have also evolved a lot over time (Shelton et al., 2015), according to some authors (Bria and Morozov, 2018) this terminology was first coined by MIT researchers in Boston. In the first decade of the 2000s, they noticed how modern cities designed around the private car and monofunctional areas had become increasingly congested, polluted and insecure; how citizens were spending more and more of their precious time commuting; how communities were breaking up more and more. Hence the awareness of the need, instead of separating the systems on the basis of functions – water, food, waste, transport, education, energy – to consider them holistically. According to MIT, rather than being focused only on access and distribution systems, our cities need dynamic, network-like, self-regulating systems that take into account complex interactions.

Since its origins, therefore, the concept of smart city has been oriented toward improving the urban environment and the quality of life of citizens, and so it continues. The focus is on the promise of a more sustainable urban environment and a radical change in the urban services’ supply system through the production and integration of urban data (Batty, 2013; March and Ribera-Fumaz, 2016; Buck and While, 2017). At the European level, for example, four years ago the European Commission (EC) established the European Innovation Partnership on Smart Cities and Communities which aims to provide a “marketplace of ideas” for smart mobility, procurement, planning etc., (European Commission, 2019). Moreover, among the last funding programs, Horizon Europe provided 5 main missions areas, one of which is dedicated to ‘Climate-neutral and Smart Cities’ (see *infra*). Today, an ever increasing number of (smart) cities label themselves as ‘inclusive’ (Townsend, 2013), recognizing the importance of citizens in the co-creation of “smart cities” (Saunders and Baeck, 2015), but playing on the ambiguity of the term participation. Indeed, it is evident that smart cities are ‘participatory’, but participated by whom^4^? There is little clarity, guidelines and proof on what people-centered “smart cities” could mean in practice (Cowley et al., 2018), as participation and inclusion do not have the same meaning (Beretta, 2016). In the present paper, by ‘inclusiveness’ we mean the extension to all citizens, therefore also to the weakest and marginalized groups of the population, of the possibility of benefiting from a good/service or of participating in some initiative/activity (Beretta, 2016). Differently, ‘participation’ simply is the act of taking part in an activity or event (Oxford dictionary).

Starting from the essential research conducted – among others – by Harvey (1985, 1996, 2000) and Sassen (1991) at the end of the last century on the theme of the inclusive capacity of our cities, and focusing on the neoliberal ethos underpinning the smart city concept (Hollands, 2008; Greenfield, 2013; Vanolo, 2013), numerous studies show that the potential for co-creating “smart” and “just” cities has not been fully realized so far (de Jong et al., 2015). Even though sustainability is at the core of the smart city discourse, its social and equity dimensions are often neglected (March, 2021). With reference to this issue, many authors highlight how the smart cities approach to social problems reflects the neoliberal rationality, focusing on market-led solutions, considering citizens above all as consumers, producers and entrepreneurs, and pushing for inclusion of the private sector (e.g., Iveson, 2011; Rifkin, 2014; Ritzer, 2015; Marvin et al., 2016; Cardullo and Kitchin, 2019b; Cuppini, 2020; Lee et al., 2020; O’Malley and Smith, 2022). Furthermore, recently the need has emerged to recognize how the neoliberal discourse and its declination in smart cities cannot be generalized but must be analyzed in the socio, political, geographical and economic context of reference (Cahill et al., 2018; Cardullo and Kitchin, 2019a, 2019b). For this reason, it is necessary to explore the different models of citizenship envisaged by Smart Cities programs, the different political and economic rationales embraced, and the specific groups with whom powerful stakeholders and decision makers do not want to share the benefits (Nam and Pardo, 2011; Marvin and Luque, 2015; O’Malley and Smith, 2022).

Other recent studies have highlighted the problem of inequalities connected to smart urbanism (Michalec et al., 2019), focusing on slightly different aspects including mobility.

Already in her study carried out in 2016, Beretta (2018) highlighted the unfairness of the localization of certain sharing services which were mostly concentrated in the most affluent, central, neighborhoods. Today, attention is directed above all to the difficulties that older people may encounter in contemporary transport systems (particularly public, but not only), characterized by a high degree of computerization (Behrendt et al., 2017; Sourbati and Behrendt, 2020), highlighting the necessity of ‘age-friendly smart mobility’ (Loos et al., 2020).

### 1.2. Urban environmental sustainability and inclusiveness

In the context of the fight against climate change, national and supranational institutions such as the European Union are focusing on greening initiatives to solve urban sustainability problems, not only with reference to the environment, but also to human beings, underlining the co-benefits in terms of health, social relationships, wellbeing.

Nevertheless, in the recent urban ecology debate, several authors are beginning to delve deeper into the issue of how to distribute benefits deriving from – for example – green infrastructures and nature-based solutions among city inhabitants, or to what extent such initiatives contribute toward reducing inequalities (*cf.* Botzat et al., 2016; De la Barrera et al., 2016a,b; Kabisch et al., 2016; Wachsmuth et al., 2016; Haase et al., 2017; Beretta, 2019; Haase, 2019). Some argue that, under certain circumstances, the way greening strategies are designed and implemented carries a paradoxical risk of producing greater inequality among social groups rather than fostering social cohesion and inclusiveness (Krueger and Gibbs, 2007; Dooling, 2009; Checker, 2011; Wolch et al., 2014; Anguelovski et al., 2018). Haase et al. (2017) remind us that undoubtedly greening cities, installing new parks and using the space along the streets for diverse greenery contributes to an increase in well-being and enhances the attractiveness of open spaces in cities. However, some studies (*cf.* Nicholls and Crompton, 2005; Conway et al., 2010; Brander and Koetse, 2011; Heckert and Mennis, 2012; Saphores and Li, 2012) have analysed real estate market price trends, finding that proximity to green areas increases house prices, whereas others (De la Barrera et al., 2016a; Luz et al., 2019) show how unequal socio-spatial distribution is reflected in different quantities, sizes, quality and structure of green areas. Poor areas generally have less vegetation, compared to more well-off areas, which are often rich in private gardens and shaded green areas providing a vast range of ecosystems’ services (De la Barrera et al., 2016b). The rise in inequalities documented across cities is reflected in an increasingly inhomogeneous distribution of assets among urban residents, for example access to urban green spaces or recreational areas, the possibility to live in a healthy place, and exposure to risks (Kabisch and Haase, 2014). Maintaining access to greenery is becoming a problem in many Eastern European cities currently experiencing a post-socialist phase, where regulation of this aspect is rather weak and, coupled with a strong will to maximize the potential of a free market, results in the transformation of numerous public green areas into private gardens (Hirt, 2012; Kronenberg, 2015).

Arguments coming from the environmental justice perspective ask for issues such as distributional, procedural and interactional justice with respect to access to and behaviour in green spaces (Wolch et al., 2014; O’Brien et al., 2017; Haase, 2019). Very often, marginalized or less privileged social groups experience difficulties in accessing green space and therefore in enjoying its benefits because of the already existing socioeconomic inequalities (e.g., Kabisch and Haase, 2014; Nesbitt et al., 2019). But also other factors (e.g., the physical quality and the lack of safety) can reduce the accessibility for particular social groups (Carmona, 2010; Biernacka and Kronenberg, 2018), for example cultural minority groups may abstain from using a green space due to its characteristics not corresponding to their needs or preferences (Kabisch and Haase, 2014; Wolch et al., 2014).

While the studies related to distributive justice were among the first to be conducted, a vast array of literature, both recent and not so recent, (Lawrence et al., 1997; Newig and Fritsch, 2009; Paloniemi et al., 2015), focus on procedural justice, showing that, in Europe, since the 1990s, the importance of participation in biodiversity governance has significantly increased. But such initiatives are all based on the conviction (or on the simple promise) that wide-scale participation in governance of biodiversity can lead to more balanced results, at the same time allowing citizens and social groups to be listened to and be involved in conservation. However, experience shows that the participatory tools cannot be considered in any case as being able to guarantee greater justice and equity; in other words it cannot be taken for granted that reaching agreements will significantly modify the power structures of governance or influence the results of the processes of participation. In this regard, Paloniemi et al. (2015, p. 331) remember that “Among others, Schlosberg (2007) and Fraser (2009) argue that the last four decades of scholarship have focused on distributive equity, while under-theorizing the related realms of recognition and political participation (Schlosberg, 2004), highlighting the need to acknowledge the structures and procedures generating injustices (Urkidi and Walter, 2011) and the socioeconomic context which defines lines of exclusion and inclusion.” As will be recalled in more detail below, in this same line of reasoning, Bulkeley et al. (2014) identify recognition of existing forms of inequality as a substantive part of the definition of environmental justice.

In other words, whereas urban planners and public officials tend to overestimate—at least in public—the benefits of community and dialog-driven approaches in urban sustainability planning, the clear line between participatory processes and increased justice is not direct, even when inclusion is intentional (Fainstein, 2010). As Anguelovski et al. (2016, 2020) show, many instances of civic participation also reveal that facilitators or designers of new green infrastructure or of climate adaptation interventions often do not allow for participation by groups or individuals who have experienced past violence, insecurity, or crime within a specific territory.

Also in the broader literature of urban political ecology (for further detail, *cf.* Beretta, 2019), capitalist globalization and the accompanying neo-liberalization of urban governance are widely accused of exacerbating socio-spatial inequality within and between cities. A concern with procedural justice focuses attention on the ways in which decisions about public spaces are made – to what extent public spaces are themselves the object of genuinely democratic and inclusive public debate in the wider urban public sphere, and to what extent such debates are captured by powerful interests or constrained by existing societal structures (Low and Iveson, 2016). For example, even if initially inclusive or initiated by historically marginalized groups, urban greening can later be appropriated by residents from higher socioeconomic status and educational backgrounds (Connolly et al., 2013; Maantay and Maroko, 2018).

Beretta (2019) define the same concept – that is, the contextual setting – as being the pre-existing social, cultural and historic conditions that influence the equity dimensions of participation, recognition and distribution.

The topic has been dealt with by diverse authors with different nuances in terms of terminology and content. McDermott et al. (2013), for example, draw from the capability theory^5^ by Nussbaum and Sen (1993), highlighting, for example, how a citizen, to be able to exercise his right to vote, must first of all be recognized as a member of the political community, and therefore possess a minimum level of education, information, stability and financial resources.

Other authors refer to the importance of the context, speaking of ‘legitimacy’ which refers to the way in which outcomes are negotiated, administered and accepted by stakeholders and encompasses issues such as ‘the recognition of stakeholders, the acknowledgement and hearing of their concerns, the participation of stakeholders in decision making, and the distribution of decision-making power’ (Paavola, 2003, pag. 8). On the subject of ‘recognition’, Corbera et al. (2007) remember that lack of recognition is strictly correlated to political and institutional hierarchies, and that the process of making decisions, and the ways of distributing the outcomes are influenced by preceding social dynamics.

From the point of view of empirical research into environmental governance and, in particular, into the strategies for the conservation of natural resources, the subject of the relevance of contextual justice (or equity) as a dimension to be studied alongside procedural and distributive justice is dealt with above all in reference to the international dimension of justice (global north vs. global south) and to the tools for the economic evaluation of nature and of ecosystems. On the contrary, some studies highlight how procedural justice is context-dependent, but the contextual dimension has not yet been particularly studied in urban settings with reference to green protected areas and nature-based solutions. In particular, as shown by Anguelovski et al. (2020), little research has been done that considers how different social groups (e.g., female, elderly, children, low-income, and minority residents) differentially attach and articulate values, preferences, and needs regarding urban nature and ecosystem services.

Fraser (2009) underlines how the diverse dimensions of the term ‘justice’ are interrelated, for which, for example, procedural justice cannot be guaranteed in a situation in which people have very diverse capabilities of participation. In this regard, the author introduces the term ‘participatory parity’ which represents the process through which standards are identified and functional definitions appropriate to the context are processed. Adopting Fraser’s framework, Bulkeley et al. (2014) argue for the development of an understanding of environmental justice which is able to capture the notion of participatory equality and identifies recognition as the base onto which matters of participation and distribution are connected. From this definition, the authors develop a three dimensional model of environmental justice, in the form of a pyramid whose four faces (distributions, procedures, rights and responsibilities) all stand on and depart from the base of recognition, highlighting how all facets of justice are interrelated and suggesting “that each must be considered in relation to one another” (Bulkeley et al., 2014, p. 34). This model, argue the authors, is suited both for analytical purposes, studying the design of policies as they are, and as a more prescriptive instrument to inform the design of climate policies also seeking justice. For both these reasons, multidimensionality and analytical capacity, in the following analysis we decided to adopt this framework as, we argue, the model best captures possible shortcomings in the underlying understanding of justice in climate policy documents. In other words, following Bulkeley et al. (2014), we adopt a definition of urban environmental justice not just as distribution of rights and responsibilities related to climate action – albeit envisioning matters of participation and inclusion rights –, but furthermore as the recognition that distributive injustices are strictly connected to cultural, historical injustices, therefore requiring specific political action in order to be recognized first.

### 1.3. Horizon Europe Mission Climate-neutral and Smart Cities

Departing from the previous literature review, which highlighted first how smartness and technology for climate neutrality do not automatically produce social equity, and secondly that understandings of equity and fairness from policy makers often lack a more profound acknowledgment of the deeper roots of inequalities which cannot be overcome through simple participation, it is deemed at this point relevant a more detailed analysis of how decarbonization and a just transition at the urban level are understood in practice in the European context. In this instance, the strategy for urban climate neutrality promoted by the European Commission through the R&I Horizon Europe framework program is identified in the Mission Climate-neutral and Smart Cities.

From the experience gained by the European Commission from the evaluation of the Framework program Horizon 2020, running from 2014 to 2020, one of the key novelties introduced in the new Horizon Europe, running 2021–2027, is the Mission approach to some of the most pressing challenges of our societies. The novel framework means that the program should create more impact through mission-orientation and citizens engagement. “EU Missions are a new way to bring concrete solutions to some of our greatest challenges. They have ambitious goals and will deliver concrete results by 2030. They will deliver impact by putting research and innovation into a new role, combined with new forms of governance and collaboration, as well as by engaging citizens.”^6^

The mission approach has been extensively documented in the European Commission report *Mission-Oriented Research & Innovation in the EU* (Mazzucato, 2018). This approach focuses on the idea that R&I, in stimulating economic growth, does not only have a scale, but it also imprints a direction to growth. According to the European Commission, the focus on directionality of innovation in the mission-oriented approach is essential to effectively tackle broadly shared social challenges through wider policy goals and sustained economic growth.^7^ Accordingly, five criteria have been identified to define the Missions, which should “be bold, inspirational and with wide societal relevance; have a clear direction: targeted, measurable and time-bound; entail ambitious but realistic actions; be cross-disciplinary, cross-sectoral and cross-actor; drive multiple, bottom-up solutions” (Mazzucato, 2019, p. 4). A particular aspect, structural to the mission approach, is the role recognized to public engagement, as the primary means to the success of the Mission.

*“Missions must be framed within challenges that are broadly agreed to be of high societal importance. This will ensure their longevity and survival across political cycles as well as contributing to their success. It will ensure that citizens can clearly see the benefits that European research and innovation in particular, and EU intervention in general, bring to their lives and communities. […] **A mission will not inspire people unless they are part of it**” (* Mazzucato, 2018*, p. 20, emphasis added).*

Following this idea, in the new Horizon Europe five Missions tackling grand societal challenges were envisioned, ranging from Adaptation to climate change, Cancer, Cities, Oceans and water to Healthy Soils, and they were launched on the 29th September 2021 (European Commission, 2021a). The Mission Climate-Neutral and Smart Cities recognizes the urgency of the challenge of climate change at the urban level and the central role that cities could play in reaching net-zero emissions through a better use of data and technology – hence the smart –, therefore envisioning the goal of climate neutrality for at least 100 European cities by 2030. According to the European Commission, the Mission should function as a test phase during which viable solutions are recognized and implemented with the active engagement of the citizenship, which will then become applicable at European scale allowing all European cities to become climate neutral by 2050, thereby supporting the ambitious climate goals of the Green Deal – to reduce emissions by 55% by 2030 and to become climate-neutral by 2050.

It is undoubtful that reaching these challenging targets will not be possible without an active role in emissions’ reduction coming from urban settlements, which, despite covering only 4% of European ground, are where 75% of the total European population resides (European Commission, 2021b). Globally, more than half of the world’s population lives in cities and urban areas, and they are expected to reach two thirds by 2050. At the same time, cities worldwide produce more than 60% of greenhouse gasses and consume more than three quarters of the world’s energy (UNDESA, 2019; IPCC, 2022). From this data alone it appears clear that cities are among the central actors around which the transition to a more sustainable model of development revolves. On the one hand, without the active participation of urban areas, every attempt at the national or international scales of governance to combat climate change would prove doomed from the start. On the other hand, if climate action and adaptation strategies will not depart from the cities in which the majority of the world’s population lives, their citizens will be more dramatically exposed and vulnerable to the risks of a changing climate. Furthermore, evidence shows that “climate impacts are felt disproportionately in urban communities, with the most economically and socially marginalized being most affected (*high confidence*)” (IPCC, 2022, p. 909). It is in this framework that the Horizon Europe Mission Cities, by also creating synergies with the Mission Adaptation, aims at transforming European cities into climate-neutral, smart and resilient spaces, through two general objectives:

1. Deliver at least 100 climate-neutral and smart cities by 2030;
2. Ensure that these cities act as experimentation and innovation hubs to enable all European cities to follow suit by 2050.^8^

These main objectives are declined in further seven specific objectives, from which the intervention logic of the Mission is structured. Without detailing all of them, from the perspective of citizen engagement and active participation, two are the main points to consider. First, Specific Objective 1 highlights the feature of the “Climate City Contract” (CCC), which is envisioned as being the core of the Mission. The CCCs will be documents^9^ signed by the cities selected by the Mission that will be co-created with all relevant stakeholder, both private actors and citizens, as well as regional and national governments, comprising three main parts: the actual Climate Neutrality Commitments, an Action Plan and a Financial Plan (Littek and Wildman, 2022). As for the mission approach in general, within the Cities Mission the role of citizen engagement is particularly stressed, as an essential feature for the success of the governance of the transition to climate neutrality (Shabb et al., 2022). Citizens and stakeholders are recognized as pivotal both as enablers of the transition through their actions and choices – concerning, e.g., energy consumption, building retrofitting and mobility practices –, as well as participants to the co-creation of policies, through their locally specific knowledge and capacities. Second, beyond SO 1, the active role of the citizenship is also stressed in Specific Objective 5 “To help cities develop, where necessary, the administrative, financial and policy capacity through innovative governance to overcome a silo approach and to ensure buy-in and commitment from citizens, local public and private stakeholders (i.e., industry, businesses) as well as regional and national authorities” (European Commission, 2021b, p. 15). As it becomes apparent in SO 5, this understanding of citizens primarily understood as enablers of the transition through their commitment (i.e., the logic of the first type of engagement presented above), recalls more what has been identified as the neoliberal ethos inhabiting the idea of the smart cities and citizens as users, entrepreneurs and/or prosumers, rather than a more comprehensive understanding of smart and just cities.

The double role played by citizen engagement for the success of the Mission is further emphasized in relation to the co-benefits that a transition to net-zero, smart cities could provide. “In particular by linking local actions for climate neutrality with some of their co-benefits such as better air quality, reduction of energy bills and road safety, it should also help develop “ownership” of the overall climate neutrality objective and thereby induce stronger local commitment and behaviour change, e.g., in mobility behaviour. These local social innovations will in turn contribute to the important process of gaining sufficient “buy-in” from local, regional, national and EU level for both the preparation and the implementation of the Climate City Contracts” (European Commission, 2021c). Also in this case, while it is undoubtable that climate change mitigation actions could bring with them co-benefits ranging from reduced pollution, better air quality, mitigation of heat island effect, which have a favorable impact on human health, it appears less clear from the available documents how social co-benefits in terms of pre-existing and arising socio-economic inequalities mitigation are accounted for. As the literature on smart cities and inclusiveness points out, the governance of smart and resilient cities by itself will not guarantee equality *per se* and, rather, might house the seeds for a further worsening of social, economic and environmental inequalities – as shown by studies on smart mobility, digital literacy as well as ecological gentrification (Dooling, 2009; Anguelovski et al., 2018; Beretta, 2019). While it is commonly accepted in the literature that environmental justice does not equate simple procedural justice, there seems to remain a belief in the official documents that participation might indeed suffice. So while we hear about co-benefits of climate neutrality, mitigation of social inequalities shall not be taken for granted as an outcome of policies that entail a reference to participation, engagement and inclusion. On the contrary, if a green and just transition is the goal, an active effort needs to be made by policy-makers at the European, national as well as urban level to integrate questions of environmental justice in the path toward climate neutrality and smartness. The research question at this point arises loud and clear: What kind of attention is given to the idea of environmental justice in the European strategy toward climate neutrality in cities? While at this stage of development of the Cities Mission a clear cut answer is not possible, what follows is a first attempt to apply a multidimensional understanding of environmental justice to the available official documents published by the European Commission supporting the implementation of the Mission.

### 1.4. The environmental justice discourse in the mission documents

As already mentioned, the Mission was launched in September 2021 with the publication of the mission Implementation Plan – one of the three main official documents published by the European Commission pertaining the Mission that will be analysed below. Soon after, the first call of expression of interest by European cities was launched, to which 377 cities from all European member states and nine associated countries responded. Of those, on the 28^th^ April 2022 the 100 selected cities were announced.^10^ Simultaneous to the launch of the Mission, also the Platform NetZeroCities was opened, a project coordinated by EIT Climate-KIC that should support the cities in the definition and implementation processes of their climate-neutrality strategies.^11^ At the moment, the selected cities together with NetZeroCities and the community of practice revolving around the Mission are in the definition stage of their Climate City Contracts. Although these CCCs will be the real litmus test for the environmental justice and equality discourse that will or will not be adopted by the cities in their road to becoming climate-neutral and smart, a first analysis of the underlying understanding of what social aspects are accounted for in the already existing documents through the lens of environmental justice will be attempted.

As it should appear clear from the previous literature review, as well as from the main programmatic European documents and policies (EGD, Communication on the Missions, etc.), the idea to evaluate the integration of environmental justice concerns in urban mitigation plans is everything but a pure stylistic exercise. The issue of justice in the debate on smart and climate-neutral cities is imperative for at least two reasons. On the one hand, it emerged clearly from the literature – to the point that the IPCC assumed the statement with a high confidence level (IPCC, 2022) –, that environmental risks are linked and impact strongly people who are already more exposed to a high risk of social exclusion. In this instance, attention to matters of recognition of pre-existing inequalities is of central importance. On the other hand, furthermore, policies designed to create smart and resilient cities that lack the proper attention to equality and justice, might even further exacerbate already existing socio-economic divides (Beretta, 2019). To acknowledge both these aspects means to realize that any climate plan and strategy needs not only integrate questions of equal opportunities of participation and distribution, in terms of rights and responsibilities, but furthermore it requires to stem from the recognition of the already existing inequalities. For this purpose, in order to properly assess the European Commission’s documents, we consider it indispensable to apply a multidimensional definition of urban environmental justice, one which also includes the dimension of justice as recognition (Fraser, 1997). As mentioned, Bulkeley et al. (2014), building on Fraser, in particular, offer a definition of environmental justice that not only includes recognition – “which views socio-economic (i.e., distributive) injustices as fundamentally linked to “cultural or symbolic injustices” which fail to give adequate recognition to certain groups (such as women, the working class, or particular racial or ethnic groups)” (p. 33) –, but it furthermore links recognition to all other facets of environmental justice (procedure, distribution, rights and responsibilities), by putting it at the base of the climate justice pyramid, in a multidimensional perspective. Their underlying assumption, shared by the authors of the present paper, is that without recognition, even the best-designed attempts to include participatory and distributive practices in urban greening and mitigation strategies will be lacking in terms of justice.

“We conceptualize climate justice as a three dimensional pyramid, which we argue better captures the multidimensional nature of what a just response to climate change might entail. We see this climate justice pyramid as diagnostic rather than prescriptive, arguing that it better captures the interdependency of distribution, procedure, rights, responsibilities and recognition, which are all facets of climate justice, and suggest the pyramid is useful both as a conceptual framework to unpack climate justice and as an analytical tool through which to shape the design of new forms of intervention” (Bulkeley et al., 2014, pp. 31-32).

Therefore, given the centrality recognized to the role of citizens for the success of the Mission, expressed through participation and co-creation, which seems to have become the undisputable mantra in the climate resilient and smart cities discourse, it is essential to understand how much of this participation is intended in terms of “winning hearts and minds” of the citizens to be on board with huge structural and transformative actions – but while also maintaining the existing interests and power structures –, and how much is indeed intended for the purpose of creating co-benefits from a socially, economically and environmentally just perspective. Otherwise “[w]ithout the explicit reference to the justice discourse, “smart cities” might become a buzzword, a term characterized by a high frequency of usage but a low potential for accountability” (Michalec et al., 2019, p. 2). With this goal in mind, the three main documents published by the European Commission as guidelines for the definition of the Mission and its implementation have been analyzed, applying the five features of environmental justice, as presented in Table 1. In detail, the official preparatory documents concerning the Cities Mission published so far are: (1) the Report by the Mission Board on the “Proposed Mission,” published as a result of the work of the Board in preparation for the launch of the Mission in September 2020, (2) the Implementation Plan by the Commission, presented concomitantly to the launch of the Mission in September 2021, and (3) the InfoKit for Cities by the Commission, which functioned as a guideline for the cities interested in responding to the first expression of interest to draft their proposals, which was published in October 2021.

**Table 1:** Reference to the different dimensions of environmental justice in the main *EC* preparatory documents for the Mission Cities.

EC Mission Cities preparatory document	Dimensions of environmental justice considered following the multidimensional definition in Bulkeley et al. (2014)				
	**Procedure**	**Distribution**	**Rights**	**Responsibilities**	**Recognition**
**(1) Proposed Mission: 100 Climate-neutral Cities by 2030 – by and for the Citizens** **Report of the Mission Board for climate-neutral and smart cities (September 2020)**	Reference present	-	-	-	-
**(2) 100 Climate-Neutral and Smart Cities** **by 2030** **Implementation Plan (September 2021)**	Reference present	Reference present	-	-	-
**(3) 100 Climate-Neutral and Smart Cities** **by 2030** **Info Kit for Cities (October 2021)**	Reference present	Reference present	Reference present	Reference present	Reference present

Author’s elaboration. See Annex 1 for the detailed reference.

## 2. Discussion

The present work has attempted to review the critical literature on Smart Cities and Urban Environmental Sustainability, in order to reach a comprehensive understanding of urban environmental justice, one that considers justice as recognition, besides distribution and participation. Once this was done, the three main preparatory documents for the Horizon Europe Mission Climate-neutral and Smart Cities have been analysed through the environmental justice framework, as developed by Bulkeley et al. (2014), in order to assess the European Commission’s understanding of the existing and arising equity issues in the path toward climate neutrality.

If we are to take a serious move to combat climate change and maintain the livability of our cities to an acceptable level, NetZero, smart cities seems to have become the only way forward for the European Union. At the same time, the transition to climate-neutrality has been recognized as an opportunity to tackle existing and arising social inequalities. But as the literature on smart and green Cities has shown, there is nothing automatic in the climate transition which will guarantee a reduction in inequalities. In particular, on the one hand, from the literature on smart cities (*cf.* par. above), we learn that more and more smart cities label themselves as ‘inclusive’, recognizing the importance of citizens in co-creation of smart cities, but playing on the ambiguity of the term participation. There is little clarity, guidelines and proof on what people-centered smart cities could mean in practice. Participation and inclusion do not have the same meaning, and it essentially because of this oversimplification that the potential for co-creating “smart” and “just” cities has not been fully realised yet. On the other hand, from the environmental justice literature (*cf.* par. above), we learned that several authors are delving deeper into the issue of how to distribute benefits deriving from greening initiatives among city inhabitants, or to what extent such initiatives contribute toward reducing inequalities. It is believed by some that under certain circumstances, greening strategies carry a paradoxical risk of deepening inequalities among already excluded social groups, rather than fostering social cohesion and inclusiveness. Even if we were to consider a purely procedural understanding of environmental justice, studies are increasingly demonstrating that participation into decision-making processes about urban greening is not enough in order to guarantee the recognition, the rights and the responsibilities to all groups equally.

With the Mission Climate-Neutral and Smart Cities, the European Commission wants to lead the way in the path toward more sustainable and livable cities through Research and Innovation that has a clear direction and that engages all citizens toward a common goal. But in order to assure that all citizens are on board and that smart cities become an opportunity for everyone, it is necessary to make sure that no citizen is left behind. To this purpose, a multidimensional understanding of environmental justice that recognizes the interconnectedness of all its dimensions (procedure, distribution, rights, responsibilities and recognition) needs to be mainstreamed across the programs, strategies, and in the specific case, the Climate City Contracts. Can we find the seeds of this acknowledgment from the official documents prepared by the European Commission so far? From this brief analysis we could surely appreciate a development in the definition and a more in-depth understanding of the role of environmental justice by the Commission, which from a “bumpy start” that saw the role of citizens mainly as enablers of the transition through their participation and engagement, has been able to expand its conception also to issues of distribution and recognition. It is in particular in the InfoKit for Cities that a clearer picture of the Commission’s commitment to environmental justice toward NetZero can be appreciated, where at least a page is completely devoted to “What can cities do to ensure a just transition?.” Far from giving a comprehensive roadmap to environmental justice for the Mission, with one page on a 100+ pages document, it nevertheless shows a more multifaceted understanding of the issue, by also recognizing the issue of justice as recognition, which should be “conceptualized as the underpinning facet [of the pyramid of environmental justice] because of its central role in relation to the other facets of justice; without recognition, for instance, true procedural justice is impossible to achieve, and distributions are likely to be affected too, whether they are distributions of rights or responsibilities” (Bulkeley et al., 2014, p. 39). Furthermore, as far as our doubts on the understanding of the role of citizens go, also the understanding of the contribution that citizen engagement could give seems more prone to an active involvement in the definition of the policies, rather than a purely “winning hearts and minds” mentality, since “engagement should aim at mobilizing the knowledge, imagination, affections and values of citizens to improve the quality of policymaking” (European Commission, 2021c, p. 64).

While the development of these documents does leave hope for a more just and equal transition to more sustainable, resilient smart cities, the Climate City Contracts will be the real test for the goodness of the environmental justice content of the NetZero transition of European Cities, leaving space for further research along their definition and implementation.

## Author contributions

IB: Draft and writing first three paragraphs. CB: Draft and writing last three paragraphs. IB and CB: Review and editing.

## Funding

Universita’ Cattolica del Sacro Cuore contributed to the funding of this research project and its publication.

## Footnotes

^2^Communication from the Commission ‘EUROPE 2020. A strategy for smart, sustainable and inclusive growth’, COM(2010) 2020.

^3^Communication from the Commission to the European Parliament, the European Council, the Council, the European economic and social committee and the Committee of the regions ‘The European Green Deal’, COM (2019) 640 final.

^4^The implementation of smartness projects tends to require the use of significant economic resources, so public administrations must open up participation in such projects to the private sector.

^5^According to their theory, capabilities are represented by the capacities, the liberty and the means needed by individuals to fully live their own existence; e.g. literacy is the capacity or skill needed to be able to read.

^6^https://research-and-innovation.ec.europa.eu/funding/funding-opportunities/funding-programmes-and-open-calls/horizon-europe/eu-missions-horizon-europe_en (last retrieved 15.02.2023).

^7^Although more and more the concept of continuous economic growth is being challenged in many instances related to sustainable development issues, also at the European Commission level. On this instance the discourse on “strong” sustainability highlights the alleged incompatibility between sustained economic growth and environmental conservation (see, e.g., Kotsila et al., 2022).

^8^Note that parallel to the European objective, the European Commission, together with the Global Covenant of Mayors for Climate and Energy and JPI Urban Europe, has launched at COP26 the global Mission Urban Transitions within the global framework of the Mission Innovation, with the goal “to mobilize 300 cities worldwide to advance their climate and energy action planning and implement systemic and innovative approaches for net-zero, resilient and people-centered transitions” (https://research-and-innovation.ec.europa.eu/news/all-research-and-innovation-news/local-action-global-impact-urban-transitions-mission-centre-starts-its-activities-2022-12-13_en last retrieved 15.02.2023).

^9^Although at the moment it is not fully clear the nature of these documents, which will not be legally binding, but rather just politically (European Commission, 2021c). For a focus on this issue see: Shabb et al. (2022).

^10^For a complete list of the 100 selected cities, from all 27 member states and 12 from Horizon Europe associated countries: https://op.europa.eu/en/publication-detail/-/publication/822ee360-c9bf-11ec-b6f4-01aa75ed71a1/language-en/format-PDF/source-256649647 (last retrieved 15.02.2023).

^11^https://netzerocities.eu (last retrieved 15.02.2023).
