# Supplementary material for: Climate-neutral and Smart Cities: a critical review through the lens of environmental justice
Source: Front Sociol. 2023 Oct 3;8:1175592. doi: 10.3389/fsoc.2023.1175592 (PMC10579794; doi:10.3389/fsoc.2023.1175592)
Supplement: Supplementary file 1 [file Data_Sheet_1.PDF]

EC Mission Cities preparatory document	Dimensions of environmental justice considered following the multidimensional definition in Bulkley et al. 2014 (text excerpt from the document in question)				
Document 1	Procedure	Distribution	Rights	Responsibilities	Recognition
(1) Proposed Mission: 100 Climate-neutral Cities by 2030 – by and for the Citizens Report of the Mission Board for climate-neutral and smart cities (September 2020)	A holistic and transformative mission for climate neutral cities, based on citizen participation and social inclusiveness, can contribute and help EU progress towards multiple SDGs (p. 9).<br><br>A meaningful engagement of citizens is therefore necessary throughout all stages of the policy cycle, from the identification and conceptualisation of Missions to their implementation, deployment and impact assessment. To be effective, citizen engagement has to be inclusive, deliberative, and influential. These three basic criteria put it in a different league compared to other ways in which we reach out to society such as communication, public consultations or stakeholder debates (p.26).	-	-	-	-

Document 2	Procedure	Distribution	Rights	Responsibilities	Recognition
(2) 100 Climate-Neutral and Smart Cities by 2030 Implementation Plan (September 2021)	The pilots will seek to address all urban systems, including mobility, energy systems and the built environment, material and resource flows, natural areas, cultural/social/financial/institutional systems, and accessible public spaces. A twinning programme will be developed in order to open up to different cities, foster exchange, facilitate replicability of the R&I pilots and build inclusive participation in effective climate action (p. 23).<br><br>The Mission Platform will help cities participating in the Mission to explore innovative governance methods. Such innovative methods should include the involvement of local key stakeholders such as civil society platforms to engage with citizens and actively involve them to develop, implement and monitor progress of the CCC. This will reduce the “silo mentality” that causes fragmentation even at local level and build inclusiveness, trust and legitimacy of the necessary actions (p. 28).<br><br>Achieving the purpose of the Mission must be a process inspired by and with the full involvement of informed citizens as well as local businesses and other economic actors that are key partners in effective local governance. This will help to build consensus that a ‘just transition’ to climate neutrality is necessary, possible and beneficial, and everyone has a role to play (p. 48).	The Just Transition Fund will complement these cohesion policy efforts. With its EUR 19.2 billion, the Fund will provide targeted support to territories facing serious socio-economic challenges in moving towards climate neutrality, and will thus ensure that transition to climate neutrality leaves nobody behind. The funding will be provided on the basis of Territorial Transition Plans prepared by Member states (pp. 35-36).	-	-	-

Document 3	Procedure	Distribution	Rights	Responsibilities	Recognition
(3) 100 Climate-Neutral and Smart Cities by 2030 Info Kit for Cities (October 2021)	Citizens' inclusion in policy making and governance is key to enabling sustainable urban living and an accelerated transition. For example, the co-creation of strategies to reduce GHG emissions is vital for their success as citizens can often provide new perspectives and solutions. […]Research shows that the social dimension is equally important to that of technology, stressing the need for institutional responses that are directed towards participatory formats (Rogers et al., 2008; Goedkoop et al., 2016). A socio-technological transition can occur only with citizens' support and participation (Vainio et al., 2019)(p. 63).<br><br>In the context of the Mission, citizen engagement should not just be about exploring opinions and interests, or eliciting knowledge and values, but about openly discussing matters of 'concern' and controversy (Chilvers and Kearns, 2015). […]In addition, engagement should aim at mobilising the knowledge, imagination, affections and values of citizens to improve the quality of policymaking (p. 64)<br><br>Broaden participation of society in decision making processes. Inclusive planning processes, co- creation and engaging communities from the beginning can improve immediate climate equity outcomes and enhance long-term stability of programmes by conveying relevant and culturally accessible climate information for socially and environmentally vulnerable groups, respecting existing cultural knowledge and values (p. 72)	As cities develop and implement their climate neutral plans and policies, they need to ensure these benefits are equitably distributed across society and that potential adverse impacts are recognized and mitigated early on. […] Additionally, existing inequalities (European Environment Agency, 2018) can be unintentionally exacerbated by climate policies. […]At the local level, cities must consider the potential harmful impact of national policies as well as the impact of their local policies. For instance, energy access and poverty, gender equality and equal opportunities for all, as well as questions of accessibility for persons with disabilities should be taken into account and promoted throughout the preparation and implementation of every climate action plan to ensure no one is left behind (p. 71).<br><br>***Integrate equity in policy and programs assessment and monitoring***. Including justice criteria in urban climate policies, programmes, infrastructure systems and urban design, will help the decision- making process to ensure they ultimately benefit all citizens. For instance, including procedures like the Strategic Environmental Assessment (European Commission, Strategic Environmental Assessment – SEA) for climate plans, often conducted at the local level, can highlight who will gain or lose as a result of these decisions and plans, and can help in assessing and monitoring the social impacts and positive effect of climate policies for every citizen (p. 72).	***Ensure social protections and adapt education programmes.*** Long-term strategies should include local social protections (i.e., for job losses in sectors that rely on fossil fuels) and a change in skills training and local school curricula to ensure social equity for affected communities (p.72).<br><br>***Integrate justice considerations into energy governance***. A supportive choice architecture and boosting of core competencies (such as financial and energy literacy) are key channels for the fair inclusion of citizens exposed to higher risks related to energy access in the energy transition (Della Valle and Sareen, 2020). The local authorities are well placed to identify vulnerable consumers and advise, train and help them with possible solutions to overcome energy poverty and improve living conditions in building, for example by financing investments in energy efficiency (p. 72).	Community-based projects often lack resources and capacity to fully address the persisting social problems of inequalities, poverty, exclusion and deprivation. Alternative business models like cooperatives and social enterprises have proven to be viable. However, entering markets and competing with large traditional players with abundant financing, and often de facto monopolies, is not easy. […]the process of empowerment of local communities should become a central and indispensable element of Social Innovation. Empowerment not only in terms of access to services and goods but also in terms of capacity-building to self-manage and own the means to achieve wellbeing during the transition (pp. 68-69).<br><br>Local authorities, employers, trade unions and research and training institutions need to cooperate to effectively integrate measures for a just transition into local sustainable economic development (p. 72)	Cities should know how to identify their vulnerable groups, locate them to address their needs, and involve them in the planning process. Additionally, cities should plan to monitor socially just climate actions to ensure that the actions and policies do not worsen or create new inequalities or unintended effects. On the contrary, cities should monitor urban climate policies and interventions and make sure they ultimately benefit all citizens and especially those most vulnerable groups (p. 71).<br><br>***Mapping social vulnerability within cities.*** Finer-scale information about social vulnerability is to be used to support decision making in better targeting resources and actions, and addressing potential future increase in their vulnerability, also in relation to the implementation of climate policies (ibidem).
